# Simultaneous Determination of Tobacco Smoke Exposure and Stress Biomarkers in Saliva Using In-Tube SPME and LC-MS/MS for the Analysis of the Association between Passive Smoking and Stress

**DOI:** 10.3390/molecules29174157

**Published:** 2024-09-02

**Authors:** Hiroyuki Kataoka, Saori Miyata, Kentaro Ehara

**Affiliations:** School of Pharmacy, Shujitsu University, Okayama 703-8516, Japan

**Keywords:** passive smoking, biomarkers, cortisol, in-tube solid-phase microextraction (IT-SPME), liquid chromatography–tandem mass spectrometry (LC–MS/MS)

## Abstract

Passive smoking from environmental tobacco smoke not only increases the risk of lung cancer and cardiovascular disease but may also be a stressor triggering neuropsychiatric and other disorders. To prevent these diseases, understanding the relationship between passive smoking and stress is vital. In this study, we developed a simple and sensitive method to simultaneously measure nicotine (Nic) and cotinine (Cot) as tobacco smoke exposure biomarkers, and cortisol (CRT), serotonin (5-HT), melatonin (MEL), dopamine (DA), and oxytocin (OXT) as stress-related biomarkers. These were extracted and concentrated from saliva by in-tube solid-phase microextraction (IT-SPME) using a Supel-Q PLOT capillary as the extraction device, then separated and detected within 6 min by liquid chromatography–tandem mass spectrometry (LC−MS/MS) using a Kinetex Biphenyl column (Phenomenex Inc., Torrance, CA, USA). Limits of detection (*S*/*N* = 3) for Nic, Cot, CRT, 5-HT, MEL, DA, and OXT were 0.22, 0.12, 0.78, 0.39, 0.45, 1.4, and 3.7 pg mL^−1^, respectively, with linearity of calibration curves in the range of 0.01–25 ng mL^−1^ using stable isotope-labeled internal standards. Intra- and inter-day reproducibilities were under 7.9% and 14.6% (*n* = 5) relative standard deviations, and compound recoveries in spiked saliva samples ranged from 82.1 to 106.6%. In thirty nonsmokers, Nic contents positively correlated with CRT contents (R^2^ = 0.5264, *n* = 30), while no significant correlation was found with other biomarkers. The standard deviation of intervals between normal beats as the standard measure of heart rate variability analysis negatively correlated with CRT contents (R^2^ = 0.5041, *n* = 30). After passive smoke exposure, Nic levels transiently increased, Cot and CRT levels rose over time, and 5-HT, DA, and OXT levels decreased. These results indicate tobacco smoke exposure acts as a stressor in nonsmokers.

## 1. Introduction

Passive smoking from environmental tobacco smoke, including secondhand smoke from cigarettes and exhaled smoke from smokers, and thirdhand smoke from indoor surfaces such as wallpaper, curtains, and clothing, is a serious public health concern [1,2,3,4,5,6]. It is a major risk factor for lung cancer, cardiovascular disease, and respiratory disease among nonsmokers. Passive smoking brings a 1.3-fold higher risk of developing lung cancer [7], and spouses of smokers have a 2-fold higher risk of developing lung cancer [8]. Additionally, the disgust caused by tobacco smoke exposure may induce stress, which is linked to various neuropsychiatric disorders [9] such as anxiety [10,11], depression [10,11], burnout [12], and stress adjustment disorder [13], as well as lifestyle-related diseases such as cardiovascular disease [14], eating disorders [15], and obesity [16].

Many studies have shown a relationship between smoking and the hypothalamus-pituitary-adrenal cortex system, a critical pathway involved in both stress response and nicotine (Nic) dependence [17]. Nic is known to induce cortisol (CRT) production, a stress biomarker [18]. However, the effects of passive smoking on stress remain unclear due to individual differences in susceptibility. Recently, we conducted a lifestyle questionnaire among nonsmokers and measured Nic and its metabolite cotinine (Cot) in hair, showing accumulation even in those unaware of tobacco smoke exposure [19]. We also found that nonsmokers exposed to tobacco smoke had increased levels of CRT and dehydroepiandrosterone sulfate (DHEAS) in saliva, indicating a stress response [20]. Therefore, it is necessary to measure tobacco smoke exposure biomarkers and stress-related biomarkers simultaneously to accurately assess the effects of passive smoking on stress.

Stress is mediated mainly by the hypothalamus-pituitary-adrenal cortex axis and the hypothalamus-sympathetic-adrenocortical system [21,22,23,24], with various endocrine hormones and neurotransmitters serving as biomarkers [24,25,26,27,28,29,30]. For example, endocrine steroid hormones such as CRT, testosterone (TES), DHEA, and DHAES [9,11,20,27], α-amylase [31], and chromogranin A [32] are widely used as biomarkers to estimate stress states. Mental stress fluctuates with emotions and can be acute or chronic. Dopamine (DA) causes elation, serotonin (5-HT) causes calmness, and oxytocin (OXT) causes warmth [24,25,26,30,33,34], making them useful biomarkers for relaxation states. Due to the low in vivo levels of these biomarkers, highly sensitive analytical equipment and reliable sample preparation methods are essential for precise measurement. In addition, while blood biomarker levels can quantify acute stress responses, the act of blood collection itself can be stressful. Urinary biomarkers may not reflect immediate stress due to delayed metabolic processes. In contrast, saliva collection is easy, non-invasive, and stress-free, with biomarkers transferring from blood to saliva in about 30 min. Although biomarker concentrations in saliva are lower than in blood [11,27,30,34], they are highly correlated with serum and plasma levels [27].

Other stress and relaxation assessment methods include psychological tools such as medical interviews and tests [24], but these are subjective. Objective evaluation methods based on physiological indicators such as heart rate, electroencephalography (EEG), fingertip pulse wave, blood pressure, skin temperature, sweating, and skin electrical activity are used [24,35,36,37,38,39,40,41]. Advances in sensors, software, and signal processing methods have led to miniaturized, portable devices for continuous monitoring [24,35,36,42,43,44,45]. Heart rate variability (HRV) is a noninvasive tool for assessing autonomic nervous system function and diagnosing stress [37,38,39,40,41,42,43,44,45]. HRV analysis using photoplethysmography [39,40,44,46] in wearable devices such as smartphones is convenient and inexpensive [45,46]. However, digital measurements can be temporary and influenced by various factors. In contrast, biochemical indicators provide objective and quantitative assessments of stress and relaxation through biomarker analysis in biological samples. Combining biochemical and physiological measurements can yield more accurate assessments of the effects of passive smoking.

Table 1 summarizes the characteristics of the main analytical methods previously reported for the determination of tobacco smoke exposure and stress biomarkers in saliva samples. Nic and Cot concentrations in saliva have been determined using immunological assay [47], gas chromatography–mass spectrometry (GC–MS/MS) [48], high-performance liquid chromatography (HPLC) [49], LC–mass spectrometry (LC–MS) [50], and LC–MS/MS [51,52]. Similarly, immunological methods [53,54,55], GC–MS [56], HPLC [57,58,59], and LC–MS/MS [60,61,62,63,64] are commonly used to measure stress- and relaxation-related biomarkers. Commercial immunosensor, enzyme immunoassay, and enzyme-linked immunosorbent assay (ELISA) kits can detect biomarkers with high sensitivity, but they have considerable analysis times per sample. While ELISA can process many samples at high throughput, it is relatively expensive. Immunological methods also require specific antibodies and are prone to cross-reactivity, making the simultaneous analysis of multiple biomarkers difficult. HPLC methods have low sensitivity and selectivity.

GC–MS/MS methods offer excellent selectivity but require time consuming derivatization to convert analytes to volatile compounds. Therefore, LC–MS and LC–MS/MS methods, which do not require derivatization and provide excellent sensitivity and selectivity, are suitable for high-throughput analysis of these biomarkers in biological samples. However, most methods need tedious and time-consuming off-line sample preparation, such as liquid–liquid extraction (LLE) and solid-phase extraction (SPE) to remove coexisting substances in biological samples. To date, no method has been reported for the simultaneous measurement of tobacco smoke exposure biomarkers and stress- and relaxation-related biomarkers.

We previously developed an in-tube solid-phase microextraction method (IT-SPME) [65] that uses a fused silica capillary coated on the inner surface with an adsorbent as an extraction device. This method is superior to the conventional off-line methods (Table 1) of introducing compounds to LC after pretreatment with LLE or SPE, as it is simple, labor-saving, and environmentally friendly because compounds can be efficiently extracted and concentrated without the use of organic solvents in a fully automated manner and introduced to LC on-line. This method has been applied to the highly sensitive and selective automated analysis of several compounds related to smoking [19,66], stress, and relaxation [20,67,68,69,70,71] by on-line coupling with LC–MS/MS. However, these methods do not simultaneously measure tobacco smoke exposure and acute stress status.

This study aims to develop a non-invasive and sensitive analytical method for the simultaneous analysis of tobacco smoke exposure biomarkers (Nic and Cot) and stress- and relaxation-related biomarkers (CRT, DA, 5-HT, MEL, and OXT) using IT-SPME LC–MS/MS methods. Additionally, the relationship between tobacco smoke exposure and stress/relaxation responses was evaluated by analyzing these salivary biomarkers in nonsmokers and by assessing HRV and autonomic balance based on fingertip pulse wave measurements.

**Table 1:** Main analytical methods previously reported for the determination of tobacco smoke exposure and stress biomarkers in saliva samples.

Biomarker ^1^	Analytical Method ^2^	Linearity Range (ng mL^–1^)	LOD (pg mL^–1^)	LOQ (pg mL^–1^)	Precision RSD (%)	Recovery (%)	Remarks	Ref.
Nic, Cot, OH-Cot	SPE GC–MS/MS	0.5–1000	500	500	1.56–9.62	89–92	Oral fluid 0.2 mL, derivatization	[48]
Nic, Cot	HPLC–UV						Saliva 5 mL	[49]
Nic, Cot, alkaloids	IT-SPME LC–MS	0.5–20	15–40	-	0.53–4.73	83–98	Saliva 0.1–0.2 mL	[50]
Nic, Cot, alkaloids	SPE LC–MS/MS	1–100	250–1000	1000	≤10	80–119	Oral fluid 0.5 mL	[51]
CRT	Immuno FET sensor	0.01–15	5		≤10	104	Saliva 0.05 mL	[55]
CRT, CRN	SPE HPLC–UV	2.0–40	36–72		2.7–7.0	88–99	Saliva 0.05 mL	[57]
CRT, CRN	IL-DDDME LC–UV/Vis	5–500	110–160	370–540	2.8–5.5	83–116	Saliva 1–1.5 mL	[58]
CRT, CRN, corticosterone	MEPS-HPLC–DAD	5–100	1500	5000	2.6–4.9	82–86	Saliva 0.4 mL	[59]
CRT, CRN, MEL	TF LC–MS/MS	0.2–10 0.001–0.1 (MEL)	-	200–1900 1.4 (MEL)	≤5	95–106	Saliva 0.05 mL	[60]
CRT, TES, DHEA	IT-SPME LC–MS	0.002–100	0.3–8.9	10–290	1.0–4.9	94–106	Saliva 0.1 mL	[67]
CRT, DHEA-S	LC–MS/MS	1.0–25.0	20–30	30–60	4.6–17.9	95–110	Saliva 0.4 mL	[61]
CRT, CRN	SPE UPLC–MS/MS	0.005–10	5	10–50	2–4	95–103	Saliva 0.2 mL	[62]
CRT, other steroids	SPE UPLC–MS/MS	0.005–5.0	-	50 (CRT)	7.2 (CRT)	-	Saliva 0.2 mL	[63]
CRT, TES, DHEA, DHEA-S	IT-SPME LC–MS	0.01–20	0.40–8.5	36–768	0.9–6.1	95–106	Saliva 0.05 mL	[20]
CRT, CRN, MEL	LLE UPLC–MS/MS	0.2–10 0.001–0.1 (MEL)	36–54 0.7 (MEL)	181–360 2.3(MEL)	7–14	86–99	Saliva 0.25 mL	[64]
Nic, Cot, CRT, DA, 5-HT, MEL, OXT	IT-SPME LC–MS	0.01–25	0.12–3.7	4–124	1.3–7.9	82–107	Saliva 0.05 mL	This study

^1^ CRN: cortisone; TES: testosterone; DHEA: dehydroepiandrosterone; DHEA-S: DHEA sulfate; ^2^ IL-DDDME: ionic liquid dispersive liquid–liquid microextraction; Immuno FET: immuno field-effect transistor; TF: turbulent flow; UPLC: ultra-performance LC.

## 2. Results and Discussion

### 2.1. Optimization of IT-SPME and Desorption of Biomarkers

**Figure 1:**
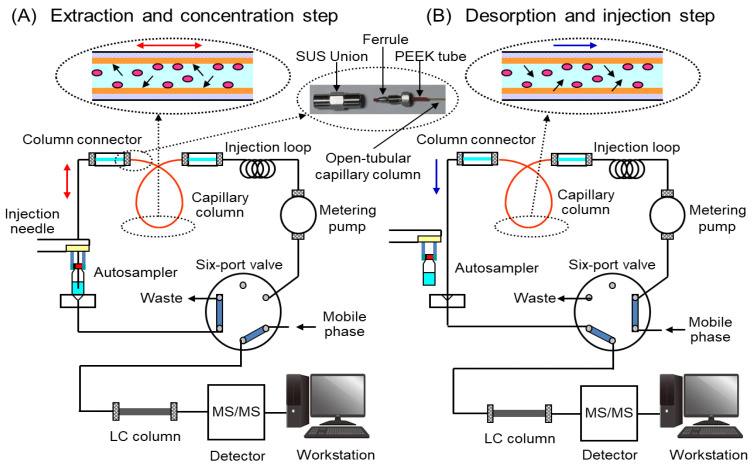
On-line IT-SPME LC–MS/MS system.

The IT-SPME system (Figure 1) is programmed with autosampler software to manage each step from extraction, concentration, and desorption of the analytes in a capillary column, to introduction into an LC separation column. This enables on-line sequential analysis of multiple samples without using organic solvents [65]. The capillary column is conditioned by washing it with methanol and water before extracting compounds from the sample solution, which prevents carryover effects from previous sample analyses. An air gap is necessary during conditioning to prevent mixing of the mobile phase and sample solution, and to facilitate desorption of analytes from the capillary coating by the mobile phase after the extraction step.

**Figure 2:**
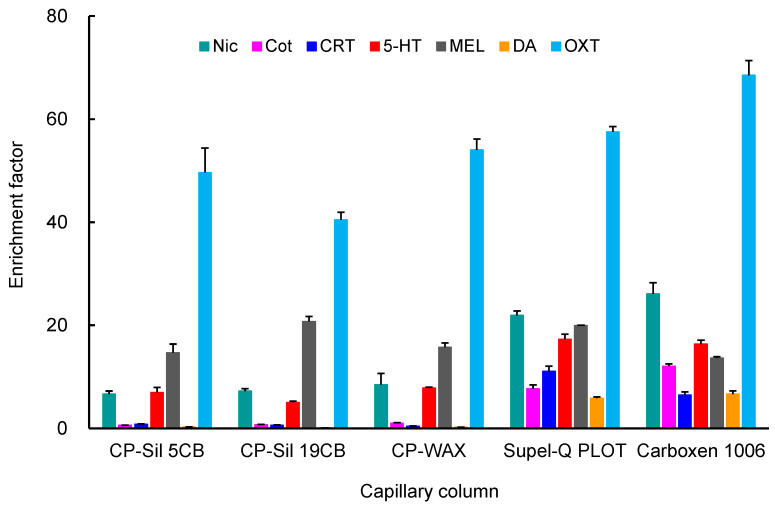
Effects of capillary coatings on IT-SPME of seven biomarkers. Extraction was performed by 25 draw/eject cycles of 40 μL of standard solution.

**Figure 3:**
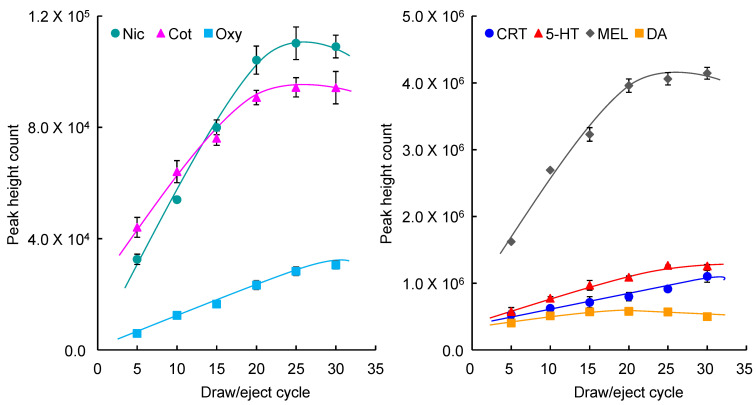
Effects of draw/eject cycles on IT-SPME of seven biomarkers. Extraction was performed with Supel-Q PLOT capillary by draw/eject cycles of 40 μL of standard solution.

To develop a simultaneous analytical method for biomarkers using on-line automated IT-SPME LC–MS/MS conditions including the length and coating type of the capillary, number, flow-rate of drawings/ejections, and pH of the sample solution were optimized using a standard mixture containing 5.0 ng mL^−1^ Nic, 1.0 ng mL^−1^ Cot, 0.2 ng mL^−1^ CRT, 2.0 ng mL^−1^ 5-HT, 1.0 ng mL^−1^ MEL, 20 ng mL^−1^ DA, and 50 ng mL^−1^ OXT. The enrichment factors of each biomarker were calculated as the peak height ratio obtained by IT-SPME compared to direct injection (10 μL) of the standard mixture. Five commercially available GC capillary columns were evaluated for biomarker enrichment. OXT showed high enrichment on all capillaries, while Nic, Cot, CRT, and DA showed lower enrichment on CP-Sil 5CB, CP-Sil 19, CB, and CP-Wax 52CB, which are coated with thin film liquid phase type adsorbents. Among the capillaries tested, Supel-Q PLOT and Carboxen 1006 (Supelco Bellefonte, PA, USA), coated with porous adsorbents, showed higher enrichment due to their large adsorption surface areas and thick films (Figure 2). Since Carboxen 1006 tends to detach its coating from the capillary inner wall, Supel-Q PLOT was chosen as the best extraction device for IT-SPME in this study. Longer capillaries increase the sample load, but they also broaden the peaks and require more time for extraction. Therefore, considering the amount of analyte extracted and peak broadening, it was optimal to use a capillary with a length of 60 cm and an inner diameter of 0.32 mm (approximately 48 μL) and a load sample of 40 μL, which does not exceed this volume. Furthermore, the number of draw/eject cycles, flow rate, and sample pH influence the amounts of compounds extracted and the extraction time. As shown in Figure 3, all seven biomarkers were efficiently extracted into the Supel-Q PLOT capillary by the repeated drawing/ejecting of 40 µL samples more than 20 times. A flow rate of 0.2 mL min^−1^ was chosen as optimum; too slow a flow rate extends extraction time, while too fast a flow rate reduces extraction efficiency. Regarding the sample solution’s pH, Nic and CRT can be efficiently extracted at a weakly acidic pH. Among the pH 3–8 buffers tested, acetate buffer (pH 4) was the most effective.

The biomarkers extracted into the stationary phase in the capillary were almost completely desorbed and introduced directly into the LC column by switching the valve and flowing the mobile phase into the capillary by column switching (Figure 1B). The absolute amount of biomarker extracted into the capillary tube was calculated by comparing the peak area obtained under optimized IT-SPME conditions using a Supel-Q PLOT capillary with 25 drawings/ejections of 40 µL standard solution at 0.2 mL min^−1^ flow rate with that obtained by direct injection (10 μL) of the standard mixture. Absolute extraction yields were 42, 49, 45, 79, 52, 36, and 40% for Nic, Cot, CRT, 5-HT, MET, DA, and OXT, respectively, but the reproducibility and quantitation of the IT-SPME method were good due to the autosampler and internal standard.

### 2.2. LC–MS/MS Analysis of Biomarkers

MS/MS operating parameters for seven biomarkers and their stable isotope-labeled compounds were optimized using API 4000 tuning software 1.6.2. These compounds showed high sensitivity in ESI positive ionization mode. The protonated ion [M + H]^+^ of each compound and the most intense fragment ion produced by cleavage of [M + H]^+^ were selected as the precursor ion (Q1 mass) and product ion (Q3 mass), respectively. The optimized parameters and MRM transitions for each compound are listed in Table 1. The results were consistent with previously reported data [19,20,50,51,52,60,61,62,63,64,66,67,68,69,70,71].

**Figure 4:**
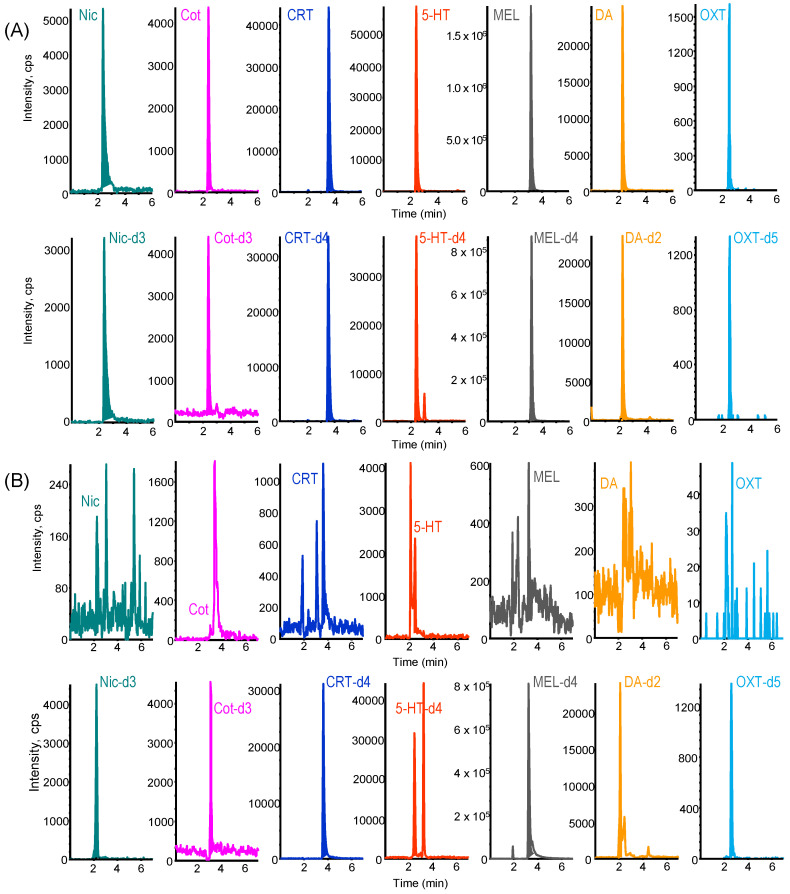
MRM chromatograms obtained from (**A**) a standard solution containing 5.0 ng mL^−1^ Nic, 1.0 ng mL^−1^ Cot, 0.20 ng mL^−1^ CRT, 2.0 ng mL^−1^ 5-HT, 1.0 ng mL^−1^ MEL, 20 ng mL^−1^ DA, 50 ng mL^−1^ OXT, and their stable isotope-labeled internal standard (IS) compounds and (**B**) 0.05 mL of saliva sample. IT-SPME LC-MS/MS conditions are described in the Materials and Methods section.

Chromatographic conditions were optimized using LC columns that allowed separation with short retention times, considering the matrix’s influence and peak shape. Among the LC columns tested, the Kinetex Biphenyl column (100 mm × 3.0 mm) showed good separation and peak shape for each biomarker. As shown in Figure 4A, the seven biomarkers and their stable isotope-labeled compounds were selectively detected as good peaks within 6 min at 0.05% formic acid solution/acetonitrile (60/40, *v*/*v*) and a flow rate of 0.2 mL min^−1^. With the developed on-line IT-SPME LC–MS/MS system, the analysis time per sample was approximately 24 min, and overnight operation enabled automated analysis of about 60 samples per day.

### 2.3. Validation of the Developed IT-SPME LC–MS/MS Method

To evaluate the analytical performance of the proposed method, linearity, sensitivity, and intra- and inter-day precisions were validated. As shown in Table 2, the calibration curves constructed from the peak height ratios of biomarkers to each IS showed a linear relationship in the range of 0.01 to 25 ng mL^−1^ (triplicate analyses of each compound at six concentrations) with correlation coefficients above 0.9974 (*n* = 18). The LOD at signal-to-noise (*S*/*N*) ratios of 3 for each biomarker ranged 0.22–3.7 pg mL^−1^, 21–53 times more sensitive than the direct injection method (10 µL injection). The intra- and inter-day precision (RSD, %) rates were 1.3–7.9% and 2.2–14.6%, respectively, with acceptable precision for quantitative analysis (Table 3).

### 2.4. Analysis of Saliva Samples

**Figure 5:**
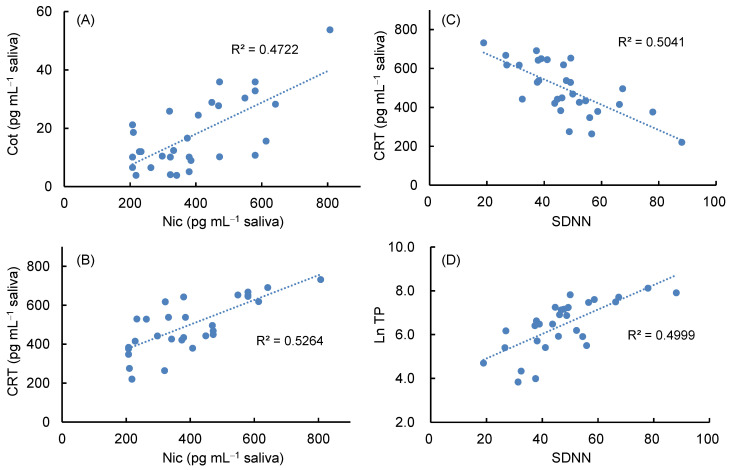
Correlation between salivary biomarker concentrations and HRV indicators in 30 nonsmokers. (**A**) Nic concentration vs. Cot concentration, (**B**) Nic concentration vs. CRT concentration, (**C**) SDNN vs. CRT concentration, (**D**) SDNN vs. Ln TP.

Saliva is a suitable biological material for noninvasive sampling, but the salivary concentrations of target biomarkers are affected by circadian rhythms [27]. In this study, saliva samples were collected with Salisoft^®^ (Assist, Tokyo, Japan) between 14:00 and 16:00, when changes in the concentrations of these biomarkers are relatively small. Saliva samples were ultrafiltrated using Amicon Ultra^®^ (Millipore, Tullagreen, Ireland) immediately after collection to remove macromolecular components. The ultrafiltrate can be stored stably in a −20 °C freezer if not analyzed immediately. The matrix effect of samples on the IT-SPME LC–MS/MS analysis could be corrected by adding the respective stable isotope-labeled IS, which behaves in ways similar to the target biomarker, to the saliva sample. As shown in Figure 5B, the salivary biomarkers were selectively detected, although background noise was noticeable in samples with low content. The LOQ (*S*/*N* = 10) of the seven biomarkers, except for OXT, were less than several tens of pg mL^−1^ saliva (Table 2), comparable to or superior to the sensitivities of previously reported LC–MS/MS methods (Table 1) [51,60,61,62,63,64]. Furthermore, the overall recoveries of biomarkers spiked to pooled saliva samples ranged from 82.1–106.6% with RSDs of 0.5–5.7% (Table 4). These results demonstrate that the proposed IT-SPME LC–MS/MS method is capable of accurately and quantitatively analyzing saliva samples.

### 2.5. Analysis of the Relationship between Stress and Tobacco Smoke Exposure by Measurements of Salivary Biomarkers and HRV

Salivary biomarkers were measured in thirty nonsmokers using the developed method, and HRV analysis was performed simultaneously by fingertip pulse wave measurement using TAS9VIEW. As shown in Table 5, despite non-smoking participants, Nic was detected in saliva at an average of 395 pg mL^−1^, and Cot was also detected at less than 1/20 of that level. These results indicate that nonsmokers are unconsciously exposed to environmental tobacco smoke [19,20,66]. In contrast, CRT, 5-HT, DA, and OXT were detected at levels ranging from tens to hundreds of pg mL^−1^, while MEL, a sleep hormone, was below the LOQ because it was a daytime saliva sample. Additionally, 5-HT was detected at a maximum level of several ng mL^−1^ with a wide range of concentrations due to dietary influences. Since salivary CRT is a known stress biomarker [9,11,27] and SDNN, LF/HF ratio, and TP in heart rate variability analysis have also been reported to be indicators of stress assessment [41,42,43], the correlation among these parameters was analyzed. As shown in Figure 5A,B, salivary Nic concentrations showed a modest positive correlation with Cot and CRT concentrations, but no significant correlation with other biomarker concentrations. In contrast, HRV analysis showed that SDNN values in the proper range (36–106) with an average of 47, and the Ln LF/Ln HF ratio averaged 1.0, indicating a good sympathetic–parasympathetic balance. As shown in Figure 5C,D, SDNN values showed a modest negative correlation with salivary CRT content and a modest positive correlation with Ln TP. These results suggest that individuals with higher SDNN levels tend to have lower CRT levels, higher levels of autonomic activity, and lower stress states. However, since salivary biomarker contents and HRV indices represent levels at the time of measurement, and there are individual differences, it is important to measure them periodically to assess fluctuations in their levels.

**Figure 6:**
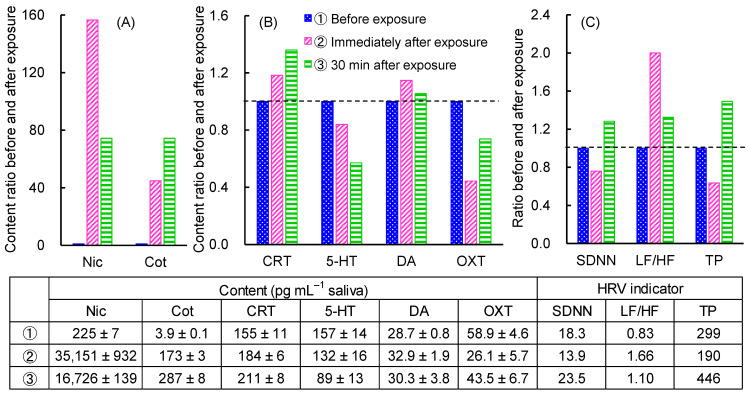
Variation in salivary biomarker concentrations and HRV indicators before and after tobacco smoke exposure in nonsmoker. (**A**) Tobacco smoke exposure biomarkers, (**B**) Stress and relaxation biomarkers and (**C**) HRV indicators.

To evaluate the effect of passive smoking on stress levels, salivary biomarker measurements and HRV analysis were performed before and after tobacco smoke exposure in one nonsmoker as a pilot study. Figure 6A,B shows the increase and decrease in salivary biomarker concentrations before and after exposure as a ratio of concentration, with the pre-exposure concentration as 1. Nic concentration increased 160-fold immediately after 30 min of tobacco smoke exposure and then halved within 30 min, while Cot concentration gradually increased. In contrast, CRT concentrations increased over time, 5-HT concentrations decreased over time, but DA concentrations increased transiently immediately after exposure. On the other hand, among the HRV indices (Figure 6C), SDNN and TP decreased transiently immediately after exposure but increased thereafter. In contrast, the LF/HF ratio increased immediately after exposure and then decreased but remained sympathetically dominant. These results suggest that exposure to tobacco smoke causes a slight increase in DA concentration with an increase in Nic concentration, but this increase cannot be sustained, resulting in an increase in CRT and a decrease in 5-HT concentration, indicating a state of stress. In contrast, HRV analysis showed a transient stress state followed by recovery. This may be because the heart rate-based stress response was observed simultaneously with tobacco smoke exposure, whereas the biomarkers were secreted later after the stress stimulus [27]. The variation of these parameters due to passive smoking confirms the modest correlations between each parameter in Figure 5.

## 3. Materials and Methods

### 3.1. Reagents and Materials

**Figure 7:**
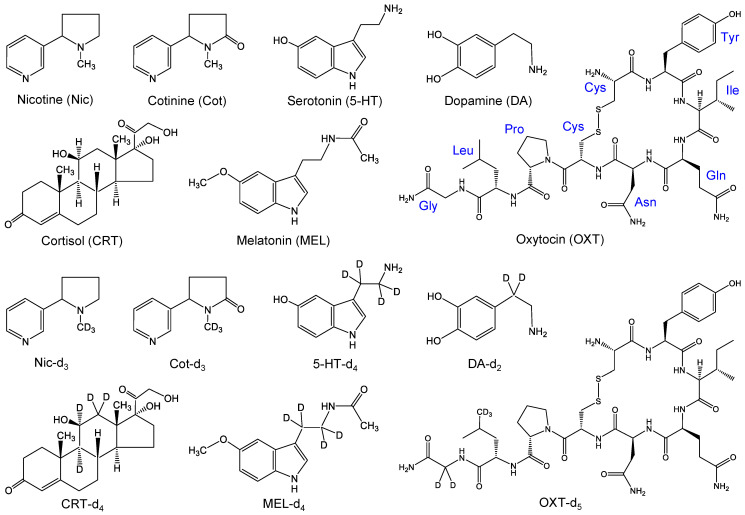
Structures of target biomarkers and their stable isotope-labeled internal standards.

Nic and its metabolite Cot were purchased from Sigma-Aldrich Japan (Tokyo, Japan); CRT, 5-HT, MEL, and DA from Sigma-Aldrich Japan (Tokyo, Japan); and OXT from Peptide Institute Inc. (Osaka, Japan). Internal standards (IS) used were Nic-d_3_ (isotopic purity 98.4%), Cot-d_3_ (isotopic purity 99.9%), CRT-d_4_ (isotopic purity 99%), MEL-d_4_ (isotopic purity 98.7%), DA-d_2_ hydrochloride (isotopic purity 99.5%), and OXT-d_5_ trifluoroacetate (isotopic purity 98.9%) from Toronto Research Chemicals Inc. (Toronto, ON, Canada), and 5-HT-d_4_ (isotopic purity >99%) from Cayman Chemical (Ann Arbor, MI, USA). Structures of these biomarkers and their stable isotope-labeled IS compounds are shown in Figure 7.

Each standard and IS except OXT and OXT-d_5_ were dissolved at 0.5 mg mL^−1^ in LC−MS grade methanol. OXT and OXT-d_5_ were dissolved at 0.1 mg mL^−1^ in LC–MS grade distilled water. Stock solutions were tightly capped and stored at −20 °C. Working standard mixtures were prepared by diluting stock solutions with distilled water to the required concentrations before use and stored at 4 °C until use. LC–MS grade methanol, acetonitrile, and distilled water were purchased from Kanto Chemical (Tokyo, Japan).

GC capillary columns (60 cm × 0.32 mm i.d.) used as extraction devices for IT-SPME included CP-Sil 5CB (100% polydimethylsiloxane, 5 μm film thick), CP-Sil 19CB (14% cyanopropyl phenyl methyl silicone, 1.2 μm film thick), and CP-Wax 52CB (polyethylene glycol, 1.2 μm film thick) from Varian Inc. (Lake Forest, CA); and Supel-Q PLOT (divinylbenzene polymer, 17 μm film thick) and Carboxen 1006 PLOT (Carboxen molecularsives, 15 μm film thick) from Supelco (Bellefonte, PA, USA).

### 3.2. Sampling, Preparation and Analysis of Saliva Samples

This study was approved by the ethics committee of Shujitsu University and informed consent was obtained from all participants. Saliva samples were collected from 7 healthy males and 23 healthy females aged 20–26 years. Each participant rinsed their mouth with water 30 min before saliva collection, refrained from eating or drinking, and provided approximately 1 mL of saliva in a collection tube using a Salisoft^®^ kit (Assist, Tokyo, Japan) containing a polypropylene–polyethylene swab. Saliva was donated between 14:00 and 16:00, when circadian rhythms of target biomarkers are less variable [27]. After removal of insoluble material by centrifugation at 2500× *g* for 1 min, the supernatant saliva sample was transferred into polyethylene tubes with caps, stored at −20 °C if not immediately used for analysis, and thawed spontaneously just prior to analysis. To each 0.05 mL of collected saliva sample, an aliquot of IS mixture solution and distilled water were added to make a total volume of 0.5 mL, and ultrafiltered at 15,000 rpm for 20 min using an Amicon Ultra^®^ 0.5-mL 3K regenerated cellulose 3000 molecular weight cut-off centrifugal filter device (Millipore, Tullagreen, Ireland). The filtrate (ca. 0.4 mL) was taken into a 2.0-mL autosampler vial with a septum. Then, 0.05 mL of 0.2 M acetate buffer (pH 4) was added, and the solution was made up to a total volume of 0.5 mL with distilled water. The vial was then placed in the autosampler for IT-SPME LC–MS/MS analysis. The salivary concentrations of the target biomarkers were calculated from the ratio of the peak heights of each biomarker to the IS compound using a calibration curve.

### 3.3. LC–MS/MS Conditions

An Agilent 1100 series LC (Agilent Technologies, Boeblingen Germany) coupled to an API 4000 triple quadrupole mass spectrometer (AB SCIEX, Foster City, CA, USA) was used for LC–MS/MS analysis. For LC separation, a Kinetex Biphenyl column (100 mm × 3.0 mm, particle size 2.6 μm; Phenomenex Inc., Torrance, CA, USA) and 0.05% formic acid/acetonitrile (60/40, *v*/*v*) as the mobile phase were used at a column temperature of 40 °C and a flow rate of 0.2 mL min^−1^. For electrospray ionization (ESI)-MS/MS, turbo ion spray voltage and temperature were 4200 V and 600 °C, respectively. Additionally, the flow rates of the ion-source gasses GS1 and GS2 were 50 L min^−1^ and 80 L min^−1^, respectively, and the flow rates of the curtain and collision gasses were 30 L min^−1^ and 4 L min^−1^, respectively. Multiple reaction monitoring (MRM) transitions of target biomarkers and their stable isotope-labeled compounds in positive ion mode and other parameters are shown in Table 6. Each compound was quantified by MRM of the protonated precursor molecular ions [M + H]^+^ (Q1) and their related product ions (Q3). LC–MS/MS data were processed using Analyst Software 1.6.2 (AB SCIEX).

### 3.4. IT-SPME Procedure and On-Line Coupling with LC–MS/MS

As shown in Figure 1, IT-SPME, using a capillary column as the extraction device, is incorporated into the on-line coupling system with LC–MS/MS. Both ends of a GC capillary (60 cm × 0.32 mm i.d. d.; 48 μL internal volume) were threaded through a 2.5 cm sleeve of a 1/16-inch polyetheretherketone tube with a 330 μm internal diameter, and a standard 1/16-inch stainless-steel nut, ferrule, and stainless-steel union connector were used to connect the injection needle and the injection loop of the autosampler. The injection loop was kept in the system to avoid contamination of the metering pump by the sample.

For extraction and concentration, a 2 mL screw cap autosampler vial with a silicone/polytetrafluoroethylene septum containing 0.5 mL of sample solution was placed in the sample tray in the autosampler. Additionally, three vials (1.5 mL methanol, 1.5 mL water, and another blank) were placed in the autosampler. Prior to sample extraction, the capillary column was washed and conditioned with two repeated drawings/ejections of 40 μL methanol, by drawing 50 µL air from the blank vial, and with two repeated drawings/ejections of 40 μL distilled water at each flow rate of 0.2 mL min^−1^ with the 6-port valve in the LOAD position (Figure 1A). The target biomarkers and the IS compounds in the sample were then extracted onto the capillary coating with 25 repeated drawings/ejections of 40 μL samples at a flow rate of 0.2 mL min^−1^ with the 6-port valve in the LOAD position (Figure 1A). After extraction, the tip of the injection needle was washed with one drawing/ejection of 2 μL methanol from another autosampler vial. For desorption and injection, the extracted compounds in the capillary column were desorbed with the mobile phase by valve switching to the INJECT position (Figure 1B), transported to the LC column, and detected by the MS/MS system. These IT-SPME steps including conditioning, extraction, desorption, and injection were fully automated by the autosampler software (Analyst 1.6.2, AB SCIEX).

### 3.5. Method Validation Study

The linearity, limit of detection (LOD), limit of quantification (LOQ), and precision of the developed method were evaluated. Linearities were validated by triplicate analyses of six concentrations of Nic (0.05, 0.1, 0.25, 0.5, 1.0, and 2.5 ng mL^−1^), six concentrations each of Cot and MEL (0.01, 0.02, 0.05, 0.1, 0.2, and 0.5 ng mL^−1^), six concentrations each of CRT and DA (0.2, 0.4, 1.0, 2.0, 4.0, and 10 ng mL^−1^), six concentrations of 5-HT (0.02, 0.04, 0.1, 0.2, 0.4, and 1.0 ng mL^−1^), and six concentrations of OXT (0.5, 1.0, 2.5, 5.0, 10, and 25 ng mL^−1^) in the presence of the IS mixture containing 1.0 ng mL^−1^ Nic-d_3_, 0.2 ng mL^−1^ Cot-d_3_, 4.0 ng mL^−1^ CRT-d_4_, 0.4 ng mL^−1^ 5-HT-d_4_, 0.2 ng mL^−1^ MEL-d_4_, 4.0 ng mL^−1^ DA-d_2,_ and 10 ng mL^−1^ OXT-d_5_. Calibration curves were constructed from the ratio of the peak heights of each compound to the IS compound at each concentration. The LOD and LOQ were calculated from the signal-to-noise ratio (*S*/*N*) of 3 and 10, respectively. For each analyte, the intra-day and inter-day precision was verified from five analyses using low, medium, and high concentration solutions, respectively, and expressed as relative standard deviations (RSD, %).

### 3.6. HRV Analysis by Fingertip Pulse Wave Measurement

HRV was measured using the acceleration pulse wave measuring device Pulse Analyzer Plus View (TAS9VIEW, YKC Corp., Tokyo, Japan). This device is designed to noninvasively evaluate the state of stress by placing a sensor between the index fingers of the left hand. It extracts the pulse with high accuracy from the interval peaks of pulse wave height for 150 sec at rest and analyzes changes in the pulse rate [39,44].

In time domain analysis, the RR interval of the HRV is analyzed, and the standard deviation of the normal-to-normal intervals (SDNN) is calculated. In frequency domain analysis, the very low frequency band (VLH, 0.003–0.04 Hz), the low frequency band (LH, 0.04–0.15 Hz) based on both sympathetic and parasympathetic functions, and the high frequency band (HF, 0.15–0.4 Hz) based on parasympathetic function are analyzed. The ratio of LF to HF or the ratio of the natural logarithm of each was used as an index of stress [37,40,41,42,43].

SDNN represents the degree of activity of the autonomic nervous system, with higher values indicating better physical condition. Additionally, an LF/HF or Ln LF/Ln HF ratio close to 1 indicates a state of autonomic balance and no stress, while a ratio below 1 indicates parasympathetic dominance and a relaxed state. Furthermore, the sum of the three spectral bands VLF, LF, and HF represents the total power (TP) of the RR interval variation, and a decrease in this value indicates decreased autonomic activity. These parameter indices were measured for thirty nonsmokers who provided saliva samples.

### 3.7. Tobacco Smoke Exposure Assessment by Biomarker and HRV Analyses

Salivary biomarkers and HRV were analyzed in one nonsmoker before and after passive smoking to evaluate the stress response to tobacco smoke exposure. According to a previously reported method [20], the nonsmoker was exposed for 30 min by nasal breathing to secondhand smoke generated by burning a single cigarette in a plastic smoke exposure chamber (W40 × D40 × H40 cm). Saliva samples were collected before, immediately after, and 30 min after the 30-min exposure trial. The content of tobacco smoke exposure biomarkers and stress- and relaxation-related biomarkers were measured according to the method in Section 3.5. Additionally, the fingertip pulse wave was measured using TAS9VIEW simultaneously with saliva collection, as described in Section 3.6.

## 4. Conclusions

In this study, a novel analytical method was developed to noninvasively and simultaneously measure biomarkers for tobacco smoke exposure and stress- and relaxation-related biomarkers, clarifying the relationship between passive smoking and stress. The proposed IT-SPME LC–MS/MS method can selectively analyze seven biomarkers with high sensitivity and accuracy by simply ultrafiltrating a small amount of saliva. This method is environmentally friendly, as it does not use organic solvents and is fully automated from extraction and concentration of sample solutions to separation, detection, and data analysis, enabling unattended nighttime operations and reducing labor costs.

A unique feature of this method is its ability to objectively analyze tobacco smoke exposure levels and stress levels in a single analysis using the same sample. Additionally, more accurate analysis of stress and relaxation states is possible by measuring the balance of autonomic nervous system activity based on heart rate variability in conjunction with biomarker level measurements. These analyses indicate that tobacco smoke exposure is a stressor in nonsmokers.

The biomarker analysis method developed in this study is expected to be a useful tool not only for analyzing the effects of passive smoking on stress, but also for the early diagnosis and prevention of related health issues.

## Figures and Tables

**Table 2:** Linearity and sensitivity of the IT-SPME LC–MS/MS method for biomarkers.

Biomarker	Linearity		LOD ^2^ (pg mL^−1^)		LOQ ^3^ (pg mL^−1^)
	Range (ng mL^−1^)	CC ^1^	Direct Injection	IT-SPME	IT-SPME
Nic	0.05–2.5	0.9977	7.0	0.22	7.7
Cot	0.01–0.5	0.9999	2.6	0.12	4.0
CRT	0.2–10	0.9997	23	0.78	27
5-HT	0.02–1.0	1.0000	18	0.39	13
MEL	0.01–0.5	0.9994	24	0.45	16
DA	0.2–10	0.9993	31	1.4	48
OXT	0.5–25	0.9998	138	3.7	124

^1^ Correlation coefficient (*n* = 18). ^2^ Limits of detection: pg mL^−1^ sample solution (*S*/*N* = 3). ^3^ Limits of quantification: pg mL^−1^ saliva sample (*S*/*N* = 10).

**Table 3:** Precision of the IT-SPME LC–MS/MS method for biomarkers.

Compound	Concentration (ng mL^−1^)	Precision (RSD ^1^ %), (*n* = 5)	
		Intra–Day	Inter–Day
Nic	0.25	5.7	9.5
	1	5.9	9.9
	2.5	5.8	9.4
Cot	0.05	3.4	7.3
	0.2	6.8	13.5
	0.5	3.2	5.0
CRT	1	6.1	6.7
	4	2.6	6.8
	10	1.6	3.8
5-HT	0.5	6.7	14.6
	2	3.0	5.7
	5	1.9	2.8
MEL	0.05	4.8	5.5
	0.2	3.0	2.3
	0.5	1.3	2.2
DA	1	5.0	7.8
	4	3.9	4.7
	10	3.4	2.9
OXT	2.5	7.9	8.0
	10	3.7	4.0
	25	3.6	3.7

^1^ RSD, relative standard deviation.

**Table 4:** Recoveries of biomarkers spiked into saliva samples.

Compound	Spiked (ng mL^−1^ Saliva) (ng mL^−1^ Saliva)	Recovery ± SD (%), (*n* = 3) (*n* = 3)
Nic	2.5	105.4 ± 2.5
	10	96.8 ± 0.6
	25	98.2 ± 0.9
Cot	0.5	105.4 ± 4.0
	2	99.1 ± 0.7
	5	100.4 ± 0.4
CRT	10	99.1 ± 1.4
	40	97.9 ± 4.4
	100	101.0 ± 1.2
5-HT	5	98.2 ± 2.2
	20	98.9 ± 1.5
	50	99.2 ± 2.6
MEL	0.5	96.9 ± 0.5
	2	96.2 ± 0.7
	5	102.9 ± 5.9
DA	10	82.3 ± 2.6
	40	96.7 ± 3.9
	100	82.1 ± 1.2
OXT	25	93.1 ± 3.7
	100	106.6 ± 1.1
	250	93.8 ± 2.5

**Table 5:** Salivary biomarker contents and HRV indicators in thirty nonsmokers.

	Content ^1^ (pg mL^−1^ Saliva)							HRV Indicator		
	Nic	Cot	CRT	5-HT	MEL	DA	OXT	SDNN ^3^	Ln LF/Ln HF ^4^	Ln TP ^5^
Max.	807	54	732	3706	<LOQ ^2^	224	691	88	1.7	9.1
Med.	377	12	482	164	<LOQ	44	114	47	0.9	6.6
Min.	207	4	220	23	<LOQ	<LOQ	<LOQ	19	0.7	3.8
Ave.	395	18	497	457	<LOQ	64	178	47	1.0	6.4
SD	153	12	134	792	–	46	162	15	0.2	1.2

^1^ The salivary biomarker content was obtained from the average of three independent measurements for each subject. ^2^ LOQ, limit of quantification; ^3^ SDNN, standard deviation of the normal-to-normal intervals; ^4^ LF, low frequency band, HF, high frequency band; ^5^ TP, total power.

**Table 6:** MRM transitions and setting parameters for target biomarkers and their stable isotope-labeled compounds.

Compound	Mass Transition (*m*/*z*)	DP ^1^ (V)	EP ^2^ (V)	CE ^3^ (V)	CXP ^4^ (V)
Nicotine (Nic)	163.1 → 132.1	70	10	20	10
Cotinine (Cot)	177.1 → 80.2	75	10	30	15
Cortisol (CRT)	363.0 → 120.9	70	10	30	10
Serotonin (5-HT)	177.2 → 160.2	25	5	15	3
Melatonin (MEL)	233.1 → 174.1	20	9	20	12
Dopamine (DA)	154.2 → 91.1	50	4	30	8
Oxytocin (OXT)	1008.3 → 724.5	60	9	40	12
Nic-d_3_	166.1 → 132.1	70	10	20	10
Cot-d_3_	180.1 → 80.2	75	10	30	15
CRT-d_4_	367.1 → 121.4	70	10	30	10
5-HT-d_4_	181.2 → 164.3	25	5	15	3
MEL-d_4_	237.1 → 178.1	20	9	20	12
DA-d_2_	156.2 → 93.1	50	4	30	8
OXT-d_5_	1013.3 → 724.5	60	9	40	12

^1^ Declustering potential (V). ^2^ Entrance potential (V). ^3^ Collision energy (V). ^4^ Collision cell exit potential (V).

## Data Availability

The data presented in this study are available on request from the corresponding author.
